# Positioning Technostress in the JD-R Model Perspective: A Systematic Literature Review

**DOI:** 10.3390/healthcare11030446

**Published:** 2023-02-03

**Authors:** Martina Pansini, Ilaria Buonomo, Clara De Vincenzi, Bruna Ferrara, Paula Benevene

**Affiliations:** Department of Human Sciences, LUMSA University, 00193 Rome, Italy

**Keywords:** ICT use, technostress, job demands, work-related stress, workplace health, well-being promotion strategies

## Abstract

This paper aims to describe the effects of Technostress on employees’ well-being and productivity. We adopted the Job Demands Resources Model as a theoretical framework to analyze the “Technostress” phenomenon in order to clarify whether and how technology can be considered a job demand, a job resource, or part of the effects of personal resources in the workplace. The sources search and selection process was conducted according to the PRISMA guidelines and regarded papers published from 2010 to 2022. Overall, the findings show that most selected papers consider ICT a job demand negatively affecting human behavior, thoughts, and attitudes. In contrast, some report that ICT acts as a job resource, thus reducing the impact of job demands and their physiological and psychological costs. Finally, a third category of studies does not consider the effects of ICT itself but gives more space to the interaction among ICT, the organizational context in which it is used, and the personal characteristics of ICT users. More specifically, the findings show how individual features and organizational procedures can shape the interpretations employees make about their ICT-related experiences at work and, consequently, their performance or well-being. Findings suggest that when ICT tools are strategically planned and used within organizations, they can enrich the employee experience at work, positively affecting the individual and the organizational level.

## 1. Introduction

The COVID-19 pandemic represented an opportunity for some organizations to fully exploit their innovative potential and improve the use of technology tools [1]. In a short time, connectivity levels increased, employee training was delivered through digital platforms within new digital solutions, and digital collaborative working methods were encouraged [2]. This is the case, for example, for the increasing use of digital solutions and the implementation of new remote working models, such as Flexible Work Arrangements (FWAs) or New Way of Working (NWW). These models include weekend work, shift work, overtime, annual hours contracts, part-time work, job-sharing, flextime, temporary/casual work, fixed-term contracts, and compressed workweeks. FWAs also include practices that allow employees to carry out their work outside the workplace, such as telecommuting or remote work and working from home. [3]. Their main aim is to boost employees’ freedom in planning and to improve their job conditions. It was shown that organizational flexibility positively affects organizational performance and the employees’ quality of life [4]. This flexibility is supported through the use of Information and Communication Technology (ICT) [5]. Overall, similar work practices, also called NWW, are supported by two essential factors: (i) Information and Communication Technology (ICT), which allows work and collaboration in different times and spaces; (ii) clear objectives to compensate for the lack of face-to-face interactions [6]. In this work, we will use the definition of FWAs as it covers different working conditions that require high use of technology at work.

After the COVID-19 emergency, more and more citizens work or study from home, more and more companies use digital communication to reach customers and manage industrial processes remotely, and more and more government institutions use digital technologies to keep in touch with citizens and companies [7].

Clarifying the impact of the FWAs on workers’ health and well-being has many practical implications for employers and workers because flexibility should be developed and adapted so that individual, organizational, and global needs are met. Therefore, it is crucial to understand both the costs and benefits of this evolving trend both on an organizational and a personal level [8]. In this regard, the pervasive use of ICT at work represents a controversial issue [9,10]. In fact, by exploiting the ubiquity of technologies, companies realize great benefits in terms of employees’ productivity and efficiency and the development of business processes. On the other hand, a growing body of research suggests a link between stress conditions and ICT at work [11]. One of the main ways in which ICT can contribute to stress at work is through the constant demands for attention and responsiveness that it creates, that in turn, can lead to feelings of time pressure and work overload in employees [12]. Furthermore, the extensive use of ICT may contribute to the blurring of boundaries between work and personal life [13]. Additionally, the use of ICT can also lead to feelings of isolation and disconnection from colleagues and supervisors [14]. In this regard, research started to acknowledge that ICT use has negative consequences that may harm individuals and organizations by reducing job satisfaction, organizational commitment [15], productivity [16], and performance [17].

This heterogeneity still needs systematization, despite the spreading use of ICT at work, above and beyond the COVID-19 pandemic. Systematizing our knowledge of the role of technology at work could help organizations choose how and when to introduce ICT tools and employees to manage better their ICT-related tasks and their impact on their private life.

Building on this, the review aims to deepen the knowledge of potential stress conditions precisely due to ICT at work, namely “technostress” [16].

We use the Job Demands Resources model (JD-R) as a theoretical framework, which combines job demands (source of stress), work resources (stress inhibitors) [18], and personal resources (stress inhibitors) [19]. It was applied across several occupational contexts and would likely be helpful when comparing findings from different organizational realities.

Few papers operationalized ICT dimensions or technostress as job demands or resources, and they showed that demands were negatively related to employees’ well-being and health [20] and positively to burnout in employees [21], as well as job resources were considered as technostress inhibitors [20]. Despite the fact that the JD-R model appears to be a promising theoretical framework for understanding the organizational implications of ICT use, research findings are still poor and fragmented.

Consequently, including the JD-R model in our work will allow us to consider three main roles for technostress within organizations: job demands, resources [22], and interacting with personal resources [23].

Finally, to the best of our knowledge, none have used the JD-R model as a theoretical framework to systematize the ICT issues and technostress’ contributions.

On this basis, our study has the following objectives: (1) to analyze the use of technologies considering them a FWAs’ expression, in terms of the consequences on employees’ well-being and productivity; (2) to offer a detailed description of the positive, negative, and protective effects of technostress; (3) based on the tripartite structure of the JD-R model, to understand whether and how technology can be considered a job demand, a job resource, or depending from employees’ uses and belief (personal resource).

### 1.1. Flexible Work Arrangements (FWA)

Flexible Work Arrangements (FWA) refers to a pattern of working conditions that enable employees to have an increased degree of control over when, where, and how they work. These options include working practices with high ICT use (e.g., remote working, home working) [24].

FWAs are classified according to work time, location, and task length (e.g., [4]). Studies on FWAs have shown positive outcomes for employees and firms.

FWAs allow employees to spend additional time and energy on themselves, as well as to allow employees to work together and, more recently, to protect workers’ health and safety [3]. In other words, FWAs allow employees to develop a more personalized and efficient allocation of such resources, depending on individual characteristics and needs [25]. Consistently, it is linked to health indicators (e.g., sleep habits, psychological health, somatic symptoms, absenteeism, exercise, and physical health) [4] and family needs (e.g., childcare, elderly care, school, and healthcare; [26]). Thus, the positive impact of such practices on employees’ personal life allows organizations to prevent stress conditions [25].

From an organizational perspective, FWAs are linked to reduced absenteeism and turnover, tardiness, and early quitting [3]. Without flexibility options, employees may try to improve their work-life balance by reducing the amount of work or taking sick days even when they are not [26]. In addition, FWAs are linked to profitability, productivity, profit, return on assets, equity, and investment [27].

These arrangements are predicted mainly to have predominantly positive effects on organizational performance. At the same time, several studies show mixed results, reporting the adverse effects of FWAs on individual and organizational performance [28].

For example, FWAs can encourage employees to keep working at home even after regular working hours [29], thus increasing overwork [24]. In this regard, practices such as teleworking increase the permeability of boundaries across life domains, potentially leading to work-family conflicts [24] and reducing psychological well-being and productivity [7,28]. Furthermore, there is a pervasive stigma on FWAs workers, which could reduce the likelihood of using it and create psychological distress [3]. FWAs can also be harmful when workers cannot voluntarily choose to use them (or not): employees with little or no choice about their work conditions may feel controlled and consequently less committed to the organization. Overall, without the appropriate organizational support, employees may experience adverse career outcomes and hostile behaviors from coworkers, thus considering FWAs inappropriate.

### 1.2. The Job Demands-Resources (JD-R) Model

The JD-R model assumes that employees face several work factors that could be divided into two broad categories: job demands and resources [18]. Demands are “those aspects of work that require sustained physical and psychological effort and are, therefore, associated with physiological or psychological costs” [22] p. 121). They include, for example, work overload, strict deadlines, conflicts with colleagues, and fear of losing a job [22].

On the other hand, job resources are “those aspects that are proportionate to the achievement of work-related objectives since they stimulate personal development and mitigate the associated physiological and psychological costs” ([23] p. 122). Examples of job resources are social support, control over one’s work, and receiving performance feedback [22].

Interestingly, the model shows that high job demands and low job resources contribute to burnout, while only high job resources (and not low demands) contribute to work engagement. Hence, by increasing resources, burnout is prevented, and engagement is promoted. On the contrary, reducing the demands would only affect burnout but not engagement [22].

To our knowledge, few studies have adopted the JD-R model [22] to clarify the role of technology at work.

When considering technology as a job resource, the constructs reported in the literature include ICT literacy, technical support, ICT engagement, ICT training [30], and support for innovation [31]. The main reason for these practices to be considered job resources is their preventive effect on stress due to ICT use [15,31].

On the contrary, when ICT is framed as job demand, it is operationalized as ICT learning problems, ICT monitoring, and work overload [32]. Over time, the intrusive nature of technology affects workers’ motivation and leads them to burnout [33].

The JD-R model was recently integrated with the notion of personal resources, intended as “positive self-evaluations that are linked to resiliency and refer to individuals’ sense of their ability to control and impact upon their environment successfully” [22] p. 122; [23] p. 124). While personal resources can be successfully integrated into the JD-R model, and their effects can be substantial, it is not yet clear if personal resources could act as mediators or moderators in the model [19]. To the best of our knowledge, despite introducing personal resources as a promising integration in the framework, only one study addressed their role in technology and its impact on worker well-being. Specifically, Wang et al. ([33]) include the idea of feeling confident in using technologies at work in their description of work self-efficacy, presenting the construct as a personal resource to cope with technological stress.

Several studies deal with the impact of technological stress at work. Such attention brought to coin a new term, namely “technostress”, to describe the risks of adverse conditions arising from the pervasive use of ICT tools. Given the centrality of this construct in the recent literature, the present review will investigate this phenomenon.

### 1.3. Technostress

Technostress is “a modern disease of adaptation caused by an inability to cope with new computer technologies healthily” [34] p. 16). It refers to a specific type of work stress caused by ICT [35]. From a psychological point of view, technostress is any negative effect on human behaviors, thoughts, and attitudes due to technology [15]. According to [16], technostress is composed of five “techno-stressors” (i.e., technological invasion, complexity, insecurity, uncertainty), namely dimensions that induce negative individual and workplace outcomes.

At the same time, as for any other stress condition, technostress can be differentiated as techno-eustress and techno-distress depending on how the individual evaluates a stressor. Techno-eustress is “the positive stress that individuals experiment in their use of information systems” because they are considered stimulating or exciting [36]. In contrast, techno-distress is “how and why individuals appraise information systems as a threat, experience consequent ‘bad’ stress, and are faced largely with detrimental outcomes” [36] p. 13). Consequently, [36] proposed a further distinction between challenge and hindrance technostress factors [36], influencing techno-eustress and techno-distress, respectively. The five harmful techno-stressors mentioned above are included in the second category.

Such classifications suggest that techno-stressors can lead to positive or negative outcomes. However, employees’ productivity and well-being may depend on personal and organizational characteristics.

A growing body of research has focused on the antecedent factors of technostress. Studies indicate that susceptibility to technostress may be due to different personal factors (including sociodemographic aspects such as gender, age, or relationship status) or situational and cultural factors (such as, in some societies, the social pressure of always having to be available for colleagues and supervisors) [12]. As shown by [12], this constant accessibility during work and leisure can negatively affect the work-life balance.

Another technostress antecedent seems to be the predisposition of individuals to be addicted to technology, which implies the excessive use of technological devices even for activities outside work [10,37]. On the contrary, the lack of digital literacy can decrease operational capabilities and increase technology-induced stress [37].

Regarding the outcomes of technostress, several studies have shown a negative association between technostress and productivity in employees. This association has been confirmed by early studies on technostress, as well as more recent studies. Despite the apparent paradox, the intensification of ICT and the stress experienced by employees negatively impact their performance and, consequently, their productivity [10,17,37,38]. This lower productivity also depends on the role stress and role overload experienced by employees due to increased tasks or roles beyond an employee’s ‘capability [10].

Finally, a small body of studies has focused on the protective factors of technostress, identifying that employees’ perceptions of self-efficacy towards technology can positively improve their adaptation to technologies and avoid the onset of technostress [10]. Moreover, employees’ personal qualities and abilities, such as dispositional and interpersonal mindfulness, may be configured as crucial personal antecedents to proactive coping for technostress [9].

Overall, current research on the topic does not allow for the systematization of the outcomes related to the use of technology at work. While the JD-R framework seems promising for this scope, a proper organization of technology’s positive and negative effects and an analysis of the interactions between technology and individual and organizational features have still to be provided. This review aims to fill such a gap partially.

## 2. Methods

Following the PRISMA [39] guidelines, this literature review aimed to identify articles published in peer-reviewed journals from 2010 to 2022 that were written in English. For what concerns exclusion criteria, publications other than research articles in peer-reviewed journals as well as publications that, while talking about technology, were unrelated to the work and organizational context, were considered not associated with the review’s topic.

### 2.1. Information Sources and Search Strategy

The following databases and search engines were employed for the search: EBSCOhost, PsycInfo, and Google Scholar. A number of keywords and strings were used to implement a Boolean search strategy, covering two main issues: the use of technologies within organizations and their psychosocial dimensions. The keywords were searched in the publication title or/and abstract. More in detail, the keywords used were:

Technologies

*“Modern technology” or “digitalization” or “digitalisation” or “Information communication technology” or “ICT” or “ICT use” or “organizational computer use” or” use of technology” or “flexible work arrangements” or “alternative work arrangements” or “flexible work policies” or “telework” or “remote work”*

In addition,

Psychosocial dimensions

*“Occupational health” or “occupational stress” or “psychosocial work condition” or “psychosocial risk” or “occupational health and safety” or “mental strain” or “human factor” or “psychosocial” or “stress” or “burnout” or “work environment” or “technostress” or “techno stress” or “digital stress” or “technology stress” or “techno anxiety” or “techno fatigue” or “psychosocial risks at work” or “strain”*

### 2.2. Data Collection Process

In the first stage, using the databases’ automatic tools, studies published in not academic or peer-review journals and not written in English were excluded. All references were selected from the online research platform EBSCO-host and specifically from the PsycInfo database. A further search was conducted on Google Scholar, but no further papers were identified. Furthermore, all references were gathered in the Zotero database, and duplicates were removed. This procedure allowed us to analyze the content of the papers deemed suitable manually. In the following stages, papers in which the applied methodology was not useful for this review’s purpose (e.g., theoretical position papers, literature reviews or meta-analyses, best practices) and did not include empirical research in the organizational contexts were eliminated. More specifically, empirical research that has analyzed the effects of technology use on samples other than employees (e.g., adolescents, adults, clinical patients, etc.) were also excluded. Finally, papers focusing on technology use at work also in relation to the technological challenges imposed by the COVID-19 pandemic were considered unrelated to this review.

### 2.3. Study Selection

**Figure 1:**
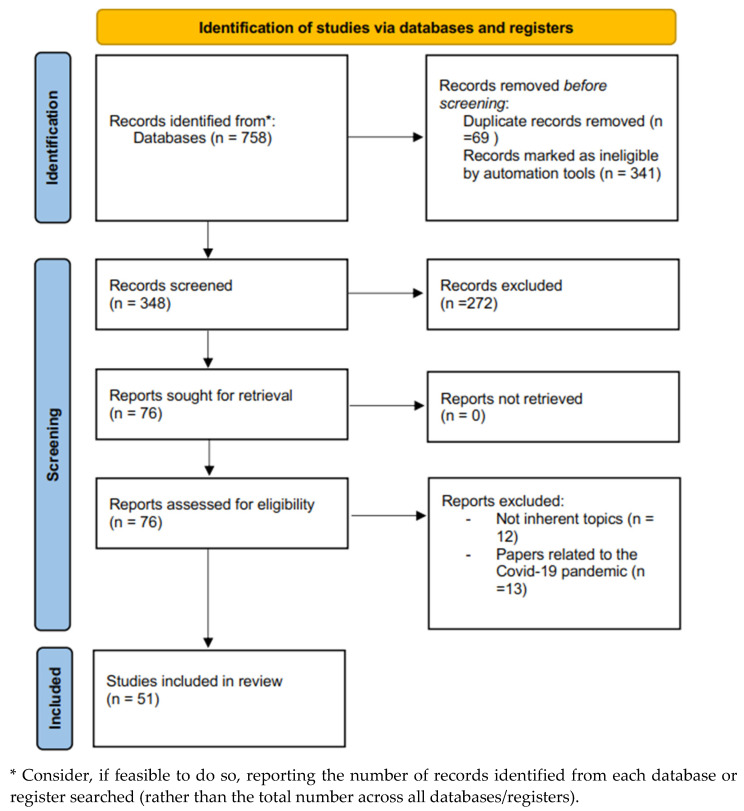
PRISMA 2020 flow diagram for new systematic reviews, which included searches of databases and registers only.

After applying the inclusive and exclusive criteria (Figure 1), 51 papers were deemed eligible and were included in the review (included papers are marked with an asterisk in the references list). In Table 1, the studies’ characteristics are described.

## 3. Results

### 3.1. Negative Factors of the Use of Technologies at Work and Their Consequences at the Individual and Organizational Level

Human-computer interaction emerged as a potential source of stress due to the always-increasing request for higher competencies in ICT use from workers, as well as limited ICT resources available from the organizations. In fact, although technology can reduce physical work and accelerate work processes, factors such as technical problems, poor usability, low situational awareness, and the need for increased qualifications are experienced by employees as potential stressors [65]. Such detrimental effects may be due to the generic use of ICT, as well as of specific devices, such as computers [47] and office-to-home smartphone use [45], and specific activities, such as using e-mail.

As expected, techno-stressors are the most reported ICT-related issues. These include *technological overload*, the information overload occurring from ICT and forcing employees to work faster and longer than usual; *technological insecurity*, the feeling of being low-skilled in the ICT field and the consequent fear of being fired; *technological uncertainty,* the role ambiguity, and task uncertainty due to fast technological changes; *technological complexity*, the inability to use technology because of malfunctions or changes in the ICT systems; t*echnological invasion or techno-invasion*, the sensation of being continuously accessible to colleagues, supervisors, or customers through technologies, and having problems with disengaging from work [21,31,48,50,57,62,64].

Techno-stressors could act as job demands [72] and negatively affect employees’ well-being [20]. Techno-insecurity and techno-invasion, indeed, are two of the major causes of burnout among employees. Regarding techno-insecurity, employees who maintain performance standards tend to spend a long time understanding and using ICT [21]. Unsurprisingly, prolonged mobile phone use is an important predictor of job burnout [42] for employees who must be in constant touch with their office or supervisors [42,51]. These behaviors generate techno-invasion and burnout because they may lead to work-family conflicts due to the overlap between work and home-life domains [57,74]. In turn, the employee perceptions of not being able to fulfill one’s roles in work and personal life contribute to turnover [29,43,73]. Furthermore, quantitative ICT demands may raise stress, depressive symptoms, and cognitive disorders such as problems with concentration, clarity of thoughts, memory, and decision-making [29,43]. Finally, employees may develop burnout due to the imposed use of ICT. The incompatibility between ICT and employees’ job beliefs (e.g., how to best provide services to customers) can exacerbate a role conflict that may lead to burnout [41].

Apart from techno-stressors, findings suggest that teleworking itself, intended as not working from one’s regular office but instead using technologies to complete work tasks and connect with the organization, may constitute a source of stress and have general adverse effects on employees’ well-being. In this regard, it has been shown that teleworking conditions are associated with higher levels of stress and psychological exhaustion when performed on a regular basis and not under the workers’ control. Consistently, a study by Kaduk showed that employees who cannot choose to telework are at higher risk for stress, burnout, intention to leave, and poor job satisfaction [63]. Similarly, higher stress levels were found in academics who use telework several times a week and those who use it less than once a month [75]. Teleworking may promote working overtime at the expense of family life [44,55,71]. Teleworking conditions may even affect the quality of relationships at work, other than in private life. Specifically, higher levels of isolation, real or perceived [49], may also affect teleworker identification with their own organization [43].

Another interesting aspect concerns workplace flexibility bias, i.e., workers’ feeling that they face career consequences for changing their schedules for family or personal reasons, which is related to minor health problems, poor self-rated health, poor sleep quality, symptoms of depression, and more frequent use of sick days [56].

Apart from psychological effects, ICT emerges as a potential cause of physical consequences, mainly related to ergonomic issues such as prolonged sitting, poor posture, ergonomically inadequate workstations, and repetitive movements [29,40,60].

Consistently, a relationship between musculoskeletal symptoms and the duration of computer use has been shown, and between the duration of mouse use and hand and arm symptoms [40]. At the same time, an employee’s persistent worry and urgency to respond quickly to work messages can lead to experiencing physical exhaustion (i.e., chronic fatigue) and poor sleep quality [29,60].

Regarding behavioral outcomes, techno-stressors negatively affect productivity and innovation on ICT-mediated tasks [31]. Techno-stressors negatively affect the user’s perception of accuracy, ease of use, timeliness, and usefulness. In addition, given the high levels of individual adaptation required for using many current and emerging ICTs, dissatisfied users may limit their use of ICT to the minimum possible levels, with detrimental consequences for their productivity.

### 3.2. Positive Outcomes of the Use of Technologies at Work

The studies about positive individual effects linked with technology use showed that contexts and personal and organizational resources might have a role in influencing the outcomes of ICT use at work. Thus, technology does not bring per se positive or negative outcomes. Rather, these depend on how the context, the personal and organizational resources, and the stressors interact among them.

From an organizational point of view, support with ICT enhances employee satisfaction [62,66]. ICT-related organizational resources, namely technical support, ICT usefulness for the job task, and involvement facilitation, lead to positive psychological responses, such as positive emotional states (i.e., hope, positive attitudes towards the tasks, and work commitment), which in turn create job satisfaction [67]. In such cases, even elements of ICT that usually foster a sense of challenge in employees (i.e., workload, time urgency, learning opportunities, job or task complexity) can generate job satisfaction as well [66].

When addressing the ICT usefulness communicated by organizations, Leung [42] showed that when employees feel that ICT could help them accomplish work tasks, bringing higher flexibility and permeability with home boundaries, it leads to higher satisfaction.

Other organizational strategies influencing such processes are: fostering employee participation in organizing and innovating the use of ICT, which brings better performances and tack crafting when using technologies [31]; higher workload related to ICT use when perceived as positively challenging instead of frustrating, which motivates employees to earn a good reputation and receive recognition for their commitment [72].

From a personal point of view, when workers perceive themselves to be autonomous and in control, they are more satisfied with their job, as they choose when and how to work. This is true even when using technologies equals working out-of-office: commuting less allows for more discretional management of time [6]. Consistently, the part-time teleworking (PTT) practice serves as a “time out” or mini-break from daily routine and interpersonal interactions with colleagues, as well as a recovery opportunity [59].

Finally, when employees are satisfied with the process and the outcomes of ICT at work, they are more likely to use ICT in the future, independently of organizational demands [48]. In this regard, Mäkiniemi et al. [70] proposed the concept of techno-work engagement, according to which the ICT use may bring an engaging condition at work. Techno-work engagement, indeed, is defined as the “positive and fulfilling well-being state or experience that is characterized by vigor, dedication, and absorption concerning the use of technology at work” (p. 2).

Studies tackling the effects of technologies at work also informed about the role of flexible work arrangements in general as well. In this regard, it was shown that, in certain conditions, remote working (and the consequent use of technologies that it requires) is linked to positive effects for employees [79]. Among the conditions that promote the positive involvement of employees in these work arrangements, we found flexibility and freedom to choose whether to opt for teleworking or not. Selected studies showed that the opportunity to adjust one’s work arrangements flexibly showed a decrease in time pressure, negative feelings at work, exhaustiveness of work as well as stress, and an increase in work satisfaction [53]. In this case, that is when workers are offered the opportunity to choose their work schedule freely, they report lower stress, lower work-to-home conflict, higher work engagement, and higher job performance [68,76], as well as higher job satisfaction and reduced psychological stress [49,52,54].

### 3.3. Protective Factors against Technostress

Individual and organizational dimensions may even buffer the effects of techno-stressors on personal outcomes. In turn, individual protective factors can be divided into personal dispositions or personality traits and coping strategies.

Regarding personality traits, Srivastava et al. [50] showed that high extraversion (a tendency to be energetic and friendly) helps employees perceive ICT use and potential techno-stressors as opportunities to increase their influence in the organization and transmit a positive self-image, thus reducing the risk for burnout. In addition, openness to experience was linked to higher awareness and likelihood to experiment with NWWs, allowing employees to perceive techno-stressors as growth opportunities. In the last case, techno-stressors are strongly linked to work engagement since employees experience greater involvement with their job. At the same time, the role of personality traits is still not clear. Other studies, indeed, suggest that extroversion may heighten, instead of buffering, the effects of ICT on burnout [64].

Regarding coping strategies related to technostress [57,72,81], the starting point is the distinction between problem-focused coping (directed at modifying the problem itself or improving the person-environment relation) and emotion-focused coping (which aims at regulating stressful emotions) proposed by Folkman et al. [82]. Studies showed that a successful problem-focused coping strategy (namely, devoting one’s resources to developing the proper skills to use ICT) builds a higher sense of control over work demands [57]. IT control, in turn, inhibits technostress, even when organizations demand the use of specific ICT products and services [58]. Shirish [77] focused on emotion-focused coping strategies, showing that when ICT use is perceived as a threat, the perception of having a good number of effective resources to counteract the strain lowers technostress. On the contrary, when ICT use is framed as an opportunity, the link between this perception and techno-strain is negative, independently of the number of affective resources. Furthermore, dysfunctional coping strategies such as moral disengagement, alcohol, and drug consumption to avoid a problem were also studied. These strategies may result in short-term cognitive and emotional relief from ICT. Nevertheless, in the long term, they may generate serious health problems and a reduced capacity for developing professional competence [72]. Furthermore, inhibitory strategies (such as venting feelings of discomfort and frustration or conversely disengaging from ICT) were shown to be irrelevant to buffer technostress [58]. Among the strategies implemented by employees, psychological detachment and control of time off also had an inhibiting effect on workplace telepressure, influencing, in turn, their satisfaction with work-life balance [13]. Finally, family and relative support has also emerged as an important protective factor: partners’ satisfaction with the work arrangement is linked with the level of stress experienced during remote work [80].

Regarding the organizational context, organizational support and social support are reported as successful coping mechanisms against technostress [31,35,48,62,69]. In addition, support from colleagues, supervisors, or managers may reduce employee exhaustion and fatigue and foster their sense of personal efficacy [35].

Other strategies that may foster employee sense of control and, thus, reduce technostress include technical support, ICT-related training, and high employee participation in ICT. These strategies can increase employee work satisfaction with ICT and their intentions to extend them through different domains of work [35,48,62]. With specific regard to training opportunities, these allow workers to feel more comfortable with new ICT and interact with qualified technical support within the organization [61]. When such training is lacking, the opposite effect was reported, with employees showing poor job performance [29,65]. In addition, providing support means clarifying boundaries between working and non-working hours, despite using technology, which may bring techno-invasion. When organizations communicate clearly about this issue, employees show higher control and balance in using work-related ICT [51]. Consistently, when employees have a good work-life balance, feel autonomous, and recognize the benefits of working out of the office, teleworking for several hours per week (vs. working mainly from the office) leads to lower stress and higher satisfaction [61].

The technology could provide a source of support (i.e., a job resource). That is the case when ICT is used to assist employees in reaching work goals and promote development, for example, by using employee autonomy, social support, and value congruence [70].

## 4. Discussion

Overall, our findings offer a broad overview of the job changes that concern social, economic, and technical-technological transformations. The use of technologies by individuals and organizations has changed radically, specifically with regard to the quantity and quality of their use. The use of flexible working practices, which aim to increase the well-being of employees and improve their performance, has also modified the vision of work and its design. Our objective was to propose a theoretical reflection, analyzing how technological innovation, and its increasingly pervasive and necessary use in work contexts, have led to the practice of flexible work arrangements (FWA), also called the modern new way of working (NWW). Despite the growing popularity of these themes, research on the impacts of FWA and NWW still provides no consistent results, and a systematic evaluation has not been conducted until now [83]. Despite such heterogeneity, the selected studies address the impact of ICT use, giving valuable suggestions on the possible consequences for workers. However, the predominance of the studies analyzed refers to the adverse effects of ICT, while positive effects are to be further explored.

Consequently, we adopted the Job Demands Resources Model as a theoretical framework to analyze the “Technostress” phenomenon. The contributions of technostress’ negative, positive, and protective effects were analyzed, identifiable as job demands and job/personal resources. In this regard, these results provide fertile ground for future meta-analyses.

### 4.1. Technology May Act as a Job Demand

Thirty-six of the selected papers present ICT as a job demand of the JDR model [18]. It shows that technostress affects human behavior, thoughts, and attitudes at the expense of employees’ physical and psychological functioning.

Consistently with this interpretation, Day et al. [30] identified a specific set of ICT demands, potentially creating technostress conditions. ICT-related demands included response expectations, hassles, employee monitoring, learning expectations, availability, poor communication, lack of ICT control, and workload. This connection is confirmed by other papers, reporting that adverse outcomes due to ICT are related to the overuse of technological devices [45,47] and the overinvolvement in ICT-based activities [46]. In other words, these studies suggest that involvement in ICT-related tasks at work may depauperate employee resources regarding, for example, a sense of control and autonomy, social support, and available time. This link is consistent with the idea of loss spirals in Hobfoll’s theory of Conservation of Resources [84], according to which people can feel stressed because of actual or potential resource loss and loss of resources due to bad resource investment. It is likely that, if not adequately addressed and organized, the use of ICT at work (both in the office and from remote working environments) may deplete employee resources, thus heightening the risk of chronic stress and burnout.

Consistently, another category of links emerging in the findings that suggest considering ICS as a job demand involves techno-creators and burnout [21,29,41,42,51,57,74]. This connection is furtherly strengthened if we consider that technostress emerges from techno-creators. Four studies reported technostress to be linked with physical [29,40,60] and psychological costs [29]. In severe cases, it can lead to high levels of physical exhaustion (i.e., a state of chronic fatigue characteristic of burnout syndrome [60]).

Other factors contributing to the ICT use-burnout link include the organizational pressures to keep up with the use of ICT, which does not allow for the necessary mental disconnection and recovery [29,44] and leads to blurring the boundaries between home and work [71,74]. Furthermore, low employee participation in ICT programming may reduce their performance [31]. Similarly, when organizational demands on how and when ICT would be used do not fit employee values, a role conflict may arise, further contributing to burnout conditions [32].

### 4.2. Technology May Act as a Job Resource

Concerning the second of the three categories of results considered above, we may interpret the positive effects of ICT on employee states as a possible indication that ICT plays a role assimilated to job resources in the JD-R model. According to this model, in addition to reducing work requests and their physiological and psychological costs, job resources direct workers to experience work engagement and involvement [18].

The first suggestion in this sense comes from the Mäkiniemi et al. [70] study, which proposed a work engagement construct tailored explicitly for online work experiences, namely techno-work engagement. Consistently with the JD-R model, techno-work engagement occurs from technology-related job resources such as autonomy, social support, and self-efficacy. While work engagement describes the feelings of vigor, dedication, and absorption of employees at work in general [18], techno-work engagement specifically refers to favorable conditions associated with using technology at work [70]. This point is consistent with one of the principles of COR theory, according to which it is required to invest resources to protect against resource loss, recover from it, and gain further resources. In turn, this principle is particularly valuable when understanding the role of ICT and how organizations use it. Mäkiniemi and colleagues’ work, indeed, showed that when ICT is framed and communicated so that employees feel empowered by its use, it becomes a source for employee well-being and productivity. Despite not referring to the work engagement construct, Tarafdar and colleagues [31] reported a similar association, showing that the higher the facilitation in using ICT from the organization, the higher the employee satisfaction with the use of ICT and, consequently, the performance at work. This finding is an example of gain spirals in the COR theory: organizations promote ICT-related resources to their employee (i.e., facilitation), who, in turn, gain other resources (i.e., satisfaction and higher performance).

Another theme emerging on the idea that ICT can improve employee lives instead of reducing their well-being is the relationship between work and personal life. In this regard, papers are frequently interested in whether and how technologies can increase work-life balance [42,48,76]. Consistently, it was shown that ICT use could play a functional role in reconciling the working sphere with the personal one. Again, this is consistent with the COR theory [84], according to which the resources gained in a particular life context (e.g., higher autonomy, more time) can benefit other life contexts (e.g., personal life), thanks to the permeability in life domains.

### 4.3. Technology Acts Differently according to Uses and Beliefs

Consistently with the frameworks suggested by Mäkiniemi et al. [70] and Tarafdar et al. [31], several studies showed that technology is not inherently resourceful or detrimental to employees’ well-being and productivity. However, organizational and personal conditions can influence the perception of technology at work. When adequately contextualized within organizations or used by skillful employees, technology can be regarded as a resource for individuals and teams. For example, Shirish [77] showed that the link between the perception of ICT, framed as an opportunity, and techno-strain is negative, independently of the results of effective resource appraisal. Again, these findings are consistent with the idea of loss and gain spirals of resources in the COR theory [84]. While in the precedent category, the role of organizational or personal resources impacting the interpretation of ICT at work was embedded in the constructs chosen by the authors (e.g., technology-related job resources in [70]), studies in this category try to explain in greater detail how usual organizational procedures (e.g., such as monitoring, support, participation) can shape the interpretation that employees make about their ICT-related experiences at work, and, consequently, their performance or well-being.

It is possible to identify organizational and individual conditions contributing to an unfruitful impact on ICT-related effectiveness and well-being at work. Regarding organizational conditions, monitoring procedures (i.e., using ICT data to verify task accuracy) may make employees feel that their personal space and autonomy are invaded. Indeed, the detrimental effects are even worse if employees believe that such data may be used against them, for example, by influencing their performance evaluation or potential job loss [30]. Regarding personal features, studies showed that coping strategies usually categorized as inhibitory strategies (e.g., venting feelings of discomfort and frustration or disengaging from ICT) cannot mitigate the detrimental effects of ICT demands and issues at work [58]. Furthermore, even employee beliefs about themselves as individuals using ICT at work can influence their well-being and performance. For example, it was shown that feelings of low effectiveness or low skills related to ICT have a role in enhancing personal stress related to the use of ICT as well as to work in general [30]. At the same time, both social and practical organizational support promotes healthy and effective ICT use for work purposes [30,31,35,48,62,69]. Regarding social support, Goetz and Boehm [69] reported that feeling supported in using one’s strengths at work allows for the buffering of insecurities due to the technologies. Specifically, Salanova et al. [35] showed that the support of colleagues reduces feelings of strain and fatigue from using technology. Regarding practical support, Fuglseth and Sørebø [48] showed that technical support, such as opening a help desk, boosts employee satisfaction with ICT and their willingness to use it more and more for work purposes.

Other dimensions promoting the positive use of ICTs at work are autonomy and job crafting opportunities, such as literacy facilitation and broader employee participation. This support can even buffer the relationships between employee expectations to improve their ICT knowledge, and stress due to the technology used [30]. Thus, having the chance to develop and grow one’s technical skills is potentially a good opportunity to boost the effects of social support [35]. Another form of facilitation includes the specific support given to HR managers when addressing employee literacy and possible conflicts emerging from using ICT at work [62]. In the latter case, organizations promoting employee autonomy in deciding how and when to use ICT for work purposes improved their sense of control over technology, their satisfaction with the job in general or with the use of technology for work purposes, and their risk of being engaged in stressful conditions because of ICT [31,48,62].

Regarding personal characteristics, employees with problem-focused coping styles tend to improve their professional skills with technology so that they feel more in control of the organizational requests [57], as well as more autonomous and effective in choosing how a specific IT can be used to fulfill the requests [58]. Even emotion-focused coping strategies can reduce techno-strain because the perception of being able to count on emotional resources lowers the perception of techno-strain itself [77]. Other personal characteristics, such as personality traits, are still receiving mixed evidence regarding their usefulness. For example, Srivastava et al. [50] reported that extraversion and openness to experience were associated with a higher likelihood of using ICT and a lower likelihood of experiencing technological stress. Khedhaouria and Cucchi [64] highlighted, instead, that extraversion was more predictive and significantly related to burnout due to technology than other personal traits.

## 5. Limitations

This review is not without limitations. First, grey literature (conference proceedings, international reports, organizational reports, doctoral dissertations) was excluded, thus potentially losing important information about ongoing studies on the topic and virtuous case studies. These sources may not have undergone the same level of peer review and quality control as the literature published in traditional academic channels, and consequently, they may not be as reliable or credible [85]. Additionally, grey literature may not be as easily accessible or discoverable as academic literature, as it is often not indexed in standard databases or search engines (e.g., SCOPUS), making it more difficult to include in a comprehensive systematic literature review [86].

Second, as contents and conclusions in systematic reviews are highly dependable on selected papers, their reliability depends on the design and execution of each study. In this case, for example, we excluded papers focusing on the technostress during the COVID-19 pandemic to analyze the stress due to ICT use without other interfering stressed sources. Indeed, we included studies with only workers’ samples, and consequently, we excluded all other participants, potentially losing results on other participant populations.

## 6. Conclusions

Flexible working arrangements have led to increased use of ICT at work. This shift has resulted in a mix of positive and negative impacts on employees’ well-being and productivity. On the one hand, technology has enabled workers to maintain work-life integrations and has improved productivity by enabling them to collaborate more effectively, regardless of their location. On the other hand, ICT use has forced employees to work longer and with a greater workload and leading them to perceive an inability to cope effectively with ICT use. Thus, in line with the first research objectives, despite flexible work arrangements have brought about changes in the use of technology, the overall impact on employee well-being and productivity remains inconclusive and requires further research.

Second, given the growing scientific interest in technological stress at work, this review focused on the topic of technostress. This last includes dimensions or “technostress creators” as technological overload, insecurity, uncertainty, complexity, and invasion. We have exhaustively described technostress ‘outcomes and highlighted that it could lead to decreased job satisfaction, increased burnout, and decreased well-being in employees. Technostress can also have positive outcomes, such as increased efficiency and productivity, improved communication and collaboration, and enhanced learning and knowledge acquisition. Technological advancements have enabled individuals to multitask, manage information, and complete tasks faster and more effectively. This can result in higher job satisfaction and a sense of accomplishment. Moreover, studies have also found that individual characteristics, such as age and personality, can affect an individual’s susceptibility to technostress.

Finally, the selected papers showed that ICTs could act both as a job demand and a job resource. As a job demand, ICT can cause technostress and negatively affect employees’ physical and psychological well-being through factors such as response expectations, employee monitoring, and overinvolvement in ICT-related activities. However, when framed and communicated by organizations as a source of employee empowerment, ICT can also act as a job resource, promoting techno-work engagement, satisfaction, and performance, as well as improving work-life integration.

However, it is interesting to observe how a category of studies emerged, not considering the effects of ICT itself but giving more space to the interaction among ICT, the organizational context in which it is used, and the personal characteristics of ICT users. Such findings suggest conclusions and practical implications for the future. The introduction of new ICT within organizations should consider the organizational context and the personal and professional characteristics of employees involved in the change. Organizations should support aware and effective ICT use while evaluating employees’ personal features that may interact with the use of ICT and its effects on professional and personal life. In this sense, technology can be a strategic tool to ensure personal performance and organizational productivity standards. It can help people improve their quality of life and work-life balance and feel more comfortable using technology.

## Figures and Tables

**Table 1:** Characteristics of included studies.

Authors/Year	Participants	Study Methodology	Technology Construct Used	Technology’s Antecedents	Technology’s Outcomes	Technology’s Mediatiors (Med) and Moderators (Mod)
Tarafdar et al., 2010 [31]	233 workers	Quantitative	Technostress	User involvement facilitation	End-users’ satisfaction; End-users’ performance	
Goldfinch et al., 2011 [40]	240 workers	Quantitative	ICT use		Stress; Musculoskeletal pain (higher ICT use); Pain (laptop, handheld devices use)	
Hennington et al., 2011 [41]	71 workers	Quantitative	Information systems (IS) use		Emotional exhaustion; Inefficacy	Role conflict (med.)
Leung, 2011 [42]	612 workers	Quantitative	ICT connectedness (ICTC)		Burnout; Job/family satisfaction	Permeability, flexibility (med., negative spillover); negative spillover (med., permeability, flexibility)
Day et al., 2012 [30]	258 workers	Quantitative	Perceived ICT demands	Hassles, expectations, availability, workload, lack of control, learning expectation, monitoring, communication (ICT demands); Personal assistance, resources support (ICT support)	Perceived ICT stress, strain, burnout	ICT Support (mod., hassles); Resource support (mod., learning expectation)
Fonner et al., 2012 [43]	193 workers	Quantitative	Connectivity paradox		Organizational Identification (−)	Stress from Interruptions (med., negative)
Golden, 2012 [44]	316 workers	Quantitative	Telework during traditional and nontraditional work hours	Time and strain-based work-to-family conflict (WFC) and family-to-work conflict (FWC)	Exhaustion (+)	Telework (mod.)
Yun et al., 2012 [45]	300 workers	Quantitative	Office-home smartphone (OHS) impact	Flexibility, productivity (OHS overload)	Job stress, resistance to OHS	Work-life conflict (med.)
Salanova et al., 2013 [35]	1072 workers	Quantitative	Technostress	Anxiety, fatigue, skepticism, self-inefficacy of ICT use (Technostrain); Excessive and compulsive ICT use, anxiety and fatigue (Techno-addiction)	Technostrain (pos. job demands; neg. job resources, personal resources); Techno addiction (pos. job demands, neg. personal resources)	
Stenfors et al., 2013 [29]	3264 workers	Quantitative	Psychosocial working conditions	Information overload, interruption by phone calls/e-mails	Cognitive complaints; depressive, sleeping problems	
Brown et al., 2014 [46]	218 workers	Quantitative	E-mail in the Workplace		Emotional exhaustion	Normative response pressure (mod., e-mail stressor); E-mail overload (med. e-mail quantity, e-mail ambiguity)
Eijckelhof et al., 2014 [47]	93 workers	Experimental	Workplace stressor		Workplace-stress	
Fuglseth and Sørebø, 2014 [48]	216 workers	Quantitative	Technostress		Extend ICT use	ICT use satisfaction (med., TC-S, TS-I)
Sok et al., 2014 [49]	418 workers	Quantitative	The work−home interface	Organizational culture (Supportive culture and innovative culture)	Positive work-home interference (WHI); Time-based; Strain-based	FWH (med. pos. Positive WHI); FWH (med. neg. time-based) FWH (med. neg. strain-based)
Srivastava et al., 2015 [50]	152 managers	Quantitative	Technostress		Work engagement, Job Burnout	Openness to experience (pos.), neuroticism (neg.) (mod., work engagement); agreeableness (pos.), extraversion (neg.) (mod., job burnout).
Ninaus et al., 2015 [51]	25 workers	Qualitative	ICT use		Better communication processes, information exchange, work-life balance; Connectivity pressure, constant availability	Connectivity behaviour awareness
Timms et al., 2015 [52]	823 workers	Quantitative	Flexible work arrangements	Organizational culture (supportive or hindrance)	work engagement, psychological strain, turnover intention	
Vesala and Tuomivaara, 2015 [53]	39 workers	Quantitative	Telework arrangement	rural work period	less time pressure, less interruptions, less negative feelings at work, less exhaustiveness of work as well as stress, increased work satisfaction	
Bentley et al., 2016 [54]	804 workers	Quantitative	Teleworker well-being	Organizational social support, telework support	psychological strain (−); job satisfaction (+)	Social isolation (med. neg. job satisfaction)
LaPierre et al., 2016 [55]	251 workers	Quantitative	Working from home		work to family conflict WFC, family to work conflict FWC	self-efficacy (mod. pos. WFC, FWC); management boundaries (mod. pos. WFC, FWC)
Nijp et al., 2016 [6]	361 workers	Qualitative	New ways of working (NWW)		More time homeworking, job satisfaction, less health	
Cech et al., 2017 [56]	2769 workers	Quantitative	Workplace flexibility bias	Workplace flexibility bias	Health problems (−); Sleep problems (−); Symptoms of depression (−); Alcohol use (−); Illness management measure (+); Stress (+); Negative work-life spillover (+)	Negative worklife spillover (med. pos. workplace flexibility bias and minor health problems, overall self-rated health, sleep, depression, and sick day use); Stress (med. pos.workplace flexibility bias and minor health problems, overall self-rated health, sleep, depression, and sick day use)
Gaudioso et al., 2017 [57]	242 workers	Quantitative	TS-C		Work-family conflict (t-invasion); job distress (t-overload)	Adaptive and maladaptive coping strategies (med.)
Pirkkalainen et al., 2017 [58]	1091 workers	Quantitative	Technostress		Strain	Distress venting (mod., neg. stressor); IT control (mod., distress venting)
Windeler et al., 2017 [59]	309 workers	Quantitative	Part-time telework (PTT)		Work exhaustion	PTT (mod., interpersonal interaction, external interaction)
Mahapatra and Pati, 2018 [21]	163 workers	Quantitative	Technostress		Burnout (t-invasion, t-insecurity)	T-invasion, t-insecurity (med., t-complexity)
Santuzzi and Barber, 2018 [60]	234 workers	Quantitative	Workplace telepressure (WPTP)		Exhaustion, poor sleep quality; Engagement	Psychological detachment (med.)
Suh and Lee, 2018 [61]	258 workers	Quantitative	Technostress	Technologies characteristics (IT complexity, IT presenteeism, pace of IT change); Job characteristics (job autonomy, task interdependence)	Strain (IT presenteeism, pace of IT change, job autonomy task interdependence); Job satisfaction (strain)	IOT (mod., all model); Work overload (med., pace of IT change, task interdependence); Invasion of privacy (med., IT presenteeism, job autonomy, task interdependence); Role ambiguity (med., pace of IT change)
Barber et al., 2019 [13]	663 workers	Quantitative	Workplace telepressure (WPTP)		Work-life balance	Psychological detachment, control, relaxation, and mastery (med.)
Florkowski, 2019 [62]	169 managers	Quantitative	HR technology, HR-staff technostress		HRT job satisfaction (HRT Support management, HR innovation climate)	HRT work stress impact (med., neg. HRT governance involvement, neg. top management HRT support, pos. HRT job insecurity impact); HRT job insecurity impact (med., neg. HR innovation climate)
Kaduk et al., 2019 [63]	758 workers	Quantative	Flexible work practices (voluntary or involuntary)	Involuntary flexible work practices	Work-to-family conflict (+), Stress (+), burnout (+), turnover intentions (+), job satisfaction (−)	
Khedhaouria and Cucchi, 2019 [64]	161 managers	Quantitative	Technostress	Agreeableness; openness to experience; extraversion; neuroticism; conscientiousness	Job burnout (low/moderate/high)	
Körner et al., 2019 [65]	36 workers	Qualitative	Stress from human-machine interaction		Technical problems, poor usability, low situation awareness, workers’ unqualified	
Benlian, 2020 [66]	115 workers	Quantitative	Technology-driven (TD) stressors		Partner satisfaction at home (pos., TCS); (neg., THS)	Negative affect (med., THS); Positive affect (med., TCS); Work-home role integration (mod., positive affect, negative affect); Organizational support (mod., positive affect, negative affect).
Califf et al., 2020 [67]	402 workers	Quantitative	Technostress		Job satisfaction; Attrition	Positive psych. response (med., TS-I); Negative psych. response (med., uncertainty, insecurity, overload).
Delanoeije and Marijke, 2020 [68]	78 workers	Experimental	Telework		lower stress, lower work-to-home conflict, higher work engagement, higher job performance on teleworking days compared to office days	
Goetz and Bohem, 2020 [69]	8019 workers	Quantitative	Technological insecurity		General health	Organizational support, friendship opportunities (mod.)
Mäkiniemi et al., 2020 [70]	729 workers	Qualitative	Technostress	Technostress; Technology’s resources (autonomy, social support, self-efficacy,)	Techno-work engagement	
Song and Gao, 2020 [71]	3962 workers	Quantitive	Work arrangement	Working at home; Working in the workplace; Bringing work at home	Happiness (−); Stress (+); Subjective well-being (−)	
Becker et al., 2021 [72]	3362 workers	Qualitative	Technostress	TS-C + interruptions, unreliability	Productivity	Exhaustion (med.); active functional-coping and dysfunctional coping (mod., exhaustion)
de Carvalho et al., 2021 [73]	473 workers	Quantitative	Technostress	TS-C (TS-C); TS-I (TS-I)	Quality of life; Intention to remain in the organization; Work-home conflict	
Hang et al., 2021 [20]	355 workers	Quantitative	Technostress	Techno-stressor	Well-being (−)	Technostress inhibitor (mod.)
Harris et al., 2021 [74]	253 workers	Quantitative	Technostress		Turnover intentions, Work-family conflict, Family burnout	Psychological entitlement (mod., t-overload-outcome, t-invasion-outcome)
Heiden et al., 2021 [75]	392 workers	Quantitative	Flexibile work	Frequency of telework	Stress (+);	
Hokke et al., 2021 [76]	4268 workers	Quantitative	Work arrangement	FLA (flexible leave arrangements); IWAFs (informal work accommodations to family)	Occupational fatigue (+); Psychological distress (+); Burnout (+); Fatigue (+)	
Shirish et al., 2021 [77]	165 managers	Quantitative	Technostress		Technostrain	TTF (med.); OTF (med.); AFFT (med., TTF-OTF)
Adamovic, 2022 [78]	604 workers	Quantitative	Telework	Power distance orientation; individualism orientation	Belief about telework effectiveness; Belief about telework isolation	Belief about telework isolation (mod., neg. telework and job stress)
Li and Wang, 2022 [79]	34,484 workers	Quantitative	Telework	Telework family initiatives	women’s mental health (+); job satisfaction (+); leisure time satisfaction (+)	
Perry et al., 2022 [80]	391 couples of workers	Quantitative	Telework	Interruptions from family during work hours	Remote work challenge stress response (−); Remote work hindrance stress response (+); Work engagement (+)	Challenge stress response (med. neg. interruptions from family during work hours and work engagement); Employee satisfaction (med. pos. interruptions from family during work hours and work engagement); Challenge stress response (med. neg. interruptions from family during work hours and spouse satisfaction with work engagement); Breaks (mod.)

## Data Availability

Not applicable.
